# Phenotypic integration mediated by hormones: associations among digit ratios, body size and testosterone during tadpole development

**DOI:** 10.1186/s12862-017-1021-0

**Published:** 2017-08-02

**Authors:** Leandro Lofeu, Renata Brandt, Tiana Kohlsdorf

**Affiliations:** Departamento de Biologia, Faculdade de Filosofia, Ciências e Letras de Ribeirão Preto, Universidade de São Paulo, Ribeirão Preto, SP 14040-901 Brazil

**Keywords:** *Leptodactylus*, Male-biased 2D:4D, Sexual dimorphism, Steroids

## Abstract

**Background:**

Developmental associations often explain phenotypic integration. The intersected hormonal regulation of ontogenetic processes fosters predictions of steroid-mediated phenotypic integration among sexually dimorphic traits, a statement defied by associations between classical dimorphism predictors (e.g. body size) and traits that apparently lack sex-specific functions (e.g. ratios between the lengths of Digits II and IV - 2D:4D). Developmental bases of female-biased 2D:4D have been identified, but these remain unclear for taxa presenting male-biased 2D:4D (e.g. *anura*). Here we propose two alternative hypotheses to investigate evolution of male-biased 2D:4D associated with sexually dimorphic body size using *Leptodactylus* frogs: I)‘*hypothesis of sex-specific digit responses*’ - Digit IV would be reactive to testosterone but exhibit responses in the opposite direction of those observed in female-biased 2D:4D lineages, so that Digit IV turns shorter in males; II) ‘*hypothesis of identity of the dimorphic digit’*- Digit II would be the dimorphic digit.

**Results:**

We compiled the following databases using *Leptodactylus* frogs: 1) adults of two species from natural populations and 2) testosterone-treated *L. fuscus* at post-metamorphic stage. Studied traits seem monomorphic in *L. fuscus*; *L. podicipinus* exhibits male-biased 2D:4D. When present, 2D:4D dimorphism was male-biased and associated with dimorphic body size; sex differences resided on Digit II instead of IV, corroborating our ‘*hypothesis of identity of the dimorphic digit’*. Developmental steroid roles were validated: testosterone-treated *L. fuscus* frogs were smaller and exhibited masculinized 2D:4D, and Digit II was the digit that responded to testosterone.

**Conclusion:**

We propose a model where evolution of sexual dimorphism in 2D:4D first originates from the advent, in a given digit, of increased tissue sensitivity to steroids. Phenotypic integration with other sexually dimorphic traits would then occur through multi-trait hormonal effects during development. Such process of phenotypic integration seems fitness-independent in its origin and might explain several cases of steroid-mediated integration among sexually dimorphic traits.

**Electronic supplementary material:**

The online version of this article (doi:10.1186/s12862-017-1021-0) contains supplementary material, which is available to authorized users.

## Background

**Fig. 1:**
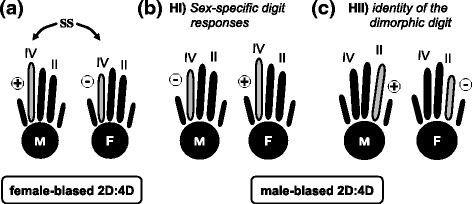
Proposed hypotheses for sexual dimorphism in 2D:4D ratios. **a** 2D:4D female-biased sexual dimorphism, which consists of larger digit ratios in females (F) than males (M), a pattern explained by Digit IV responding to sex-steroids (‘SS’, androgens and estrogens); (**b** and **c**) two hypotheses proposed here for the establishment of male-biased 2D:4D digit ratios. **b** Hypothesis I (HI): male-biased patterns are explained by inverted between-sex responses of Digit IV to sex-steroids; thus, Digit IV becomes shorter in males in response to testosterone. **c** Hypothesis II (HII): male-biased patterns reflect a change in the identity of the digit responding to sex-steroids, not the direction of the response (elongation or shortening); thus, Digit II is the one dimorphic and is longer in males and shorter in females

Sets of traits can be highly integrated due to functional and/or developmental correlations [1, 2]. Hormones likely play essential roles in the evolution of phenotypic integration because they regulate pathways involved in a multitude of developmental processes and transduce environmental signals into developmental responses [3–5]. Accordingly, correlated variation among traits might be established through indirect interactions during development mediated by the sharing of a common hormonal environment along specific developmental windows. Multiple-trait effects of hormonal variation are especially evident in sexually dimorphic phenotypes, given that sex steroids (androgens and estrogens) modulate several physiological, morphological and behavioral sex-specific features in vertebrate animals [5–7]. Such steroids may also affect the development of traits that apparently lack sex-specific functions, such as digit proportions [8, 9]. For example, the ratio between the lengths of Digit II and Digit IV (2D:4D ratio) is sexually dimorphic in several lineages [10], and apparently, differences between males and females in this feature arise from a differentiated sensitivity of specific digits to sex-steroids during development [8, 9]. Similar lengths of Digits II and IV result in higher 2D:4D ratios, while lower digit ratios occur when one digit is much longer than the other (Fig. 1) [8–11]. Historically, sexual dimorphism in the 2D:4D digit ratio has initially been identified in humans [12], and subsequently it became broadly applied as an indirect marker for detecting embryonic effects of sex-steroids on other sexually dimorphic traits, including personality, reproductive success, disease susceptibility, and sexual orientation [12–22]. This research has been mainly based on correlations among traits, with a few studies combining such correlations with experimental approaches [23–26]. Results suggest that, once the sensitivity of a given digit to hormonal levels is established, changes on the magnitude of sexual dimorphism in 2D:4D ratios likely represent conspicuous examples of phenotypic integration modulated by hormonal pleiotropy [8, 9, 11–13]. In addition to humans, an extensive record of correlations between differences in male and female 2D:4D ratios and other sexually dimorphic traits became available for mammals [8–10, 27–31], birds [10, 32–38] and lizards [10, 39–42]. Interestingly, whereas in some species the 2D:4D ratio is larger in females than males (*2D:4D female-biased,* identified in salamanders, lizards, rodents and some primates, including the chacm baboons, gorillas and some populations of chimpanzees), in other lineages the males exhibit the largest 2D:4D values (*2D:4D male-biased,* observed in anurans, birds and also some primates, including the guinea baboons and the rhesus macaque [10, 41, 43]), a variation that suggests some evolutionary lability at the level of digit tissue responses to sex steroids during development ([41, 43]; see also Fig. 1).

The developmental basis of a female-biased 2D:4D ratio has initially been identified in mice, and resides on a higher concentration of sex-steroid receptors in Digit IV than Digit II, turning the former more sensitive to sex-steroid hormones than the later (i.e., the dimorphic digit is Digit IV [8, 9]). In species characterized by a female-biased 2D:4D ratio, male Digit IV becomes longer in response to androgens (i.e., the ratio is lower), and in females, it is shorter in response to the estrogens, resulting in higher ratios ([8]; Fig. 1a). In contrast, knowledge of the mechanisms establishing a 2D:4D male-biased pattern, which has been described for example in Anura [41, 43], is comparatively much more obscure, and the identity of the dimorphic digit in this pattern remains unknown.

The arousal of sexual dimorphism in digit ratios involves the acquisition of hormone sensitivity by a given digit, and alternative scenarios might produce different dimorphic patterns (male-biased or female-biased). The developmental basis of a female-biased 2D:4D ratio has already been identified [8], and for the establishment of a 2D:4D male-biased, we foresee two alternative hypotheses (Fig. 1), which are tested here using *Leptodactylus* frogs. According to the ‘*hypothesis of sex-specific digit responses*’ (HI, Fig. 1b), a male-biased pattern would be established similarly to the 2D:4D female-biased configuration, but with Digit IV having a sexually inverse response to androgens and estrogens, which would result in a shorter Digit IV in males. Inverse responses between sexes have been described for other sexually dimorphic traits in tetrapods [6, 44], and in the 2D:4D ratio these would produce a configuration reverse to the female-biased pattern. This scenario predicts adult males having a smaller Digit IV than females and exposure to increased testosterone concentrations would result in post-metamorphic frogs presenting a shorter Digit IV than the control. Our alternative ‘*hypothesis of identity of the dimorphic digit*’ (HII, Fig. 1c) postulates that the 2D:4D male-biased pattern reflects changes in the identity of the dimorphic digit. Accordingly, Digit II, instead of IV, would respond to differences in sex steroid concentrations, becoming longer in males and shorter in females. The prediction derived from this hypothesis prognoses adult males having a longer Digit II than females, and exposure to increased testosterone concentrations during development would result in post-metamorphic frogs having a longer Digit II than the control. Given the multi-trait effects of hormonal levels during development, it is likely that in Anura the digit ratio 2D:4D is associated with body size because of phenotypic integration modulated by hormonal pleiotropy, a prediction accessed here using testosterone as a proxy for circulating sex-steroids during development.

Information on the hormonal environment of developing anurans derives mostly from studies performed with *Xenopus laevis*, which eggs present high concentrations of sex steroids hormones with maternal origin [45]. As development progresses to pro-metamorphosis stages (Gosner stages 36–40), a pattern of enzyme activity, male- and female-specific, is established, and sex steroid and steroid-receptors concentrations in specific tissues reach a peak that leads to integrated secondary sexual determination involving sexual differentiation of gonads and brain [45]; during these stages, major events of digit growth differentiation are being established [8, 61]. Modulation of phenotypic integration by testosterone is therefore expected, due to the multi-trait effects of this hormone in male development [3–7] that often include variation in body size [44]. In Anura, most species comprise female-biased body size dimorphism, a pattern likely derived from adaptive differences between sexes in life history traits [46–50]. The developmental bases for body size dimorphism in Anura remain unknown [49, 50], but it is possible that testosterone inhibits male growth in species that are female-biased for body size, as previously reported for other taxa [44]. If body size and 2D:4D ratios are phenotypically integrated through the multi-trait effects of testosterone in Anura, sexual dimorphism in the 2D:4D ratio should be coupled with variation in body size, a correlation expected both in adults from natural populations and in tadpoles raised under increased testosterone concentrations.

In this study, two databases were combined to test the hypotheses we envisage for explaining the evolution of male-biased 2D:4D ratios (Fig. 1b and c). We focus on Anura, a clade where male-biased 2D:4D ratios have been previously reported [39, 43, 51], and we use frogs from the family Leptodactylidae, a lineage where the presence and magnitude of overall sexual dimorphism vary considerably among species [52–54]. Phenotypic integration between body size and the 2D:4D ratio in Anura has also been evaluated, inferring the putative role of testosterone as a developmental regulator in the establishment of female-biased body size dimorphism in anurans. Our databases comprised 1) adults from natural populations of *Leptodactylus podicipinus* and *L. fuscus* and 2) testosterone-treated tadpoles of *L. fuscus*. While *L. podicipinus* has been described as clearly dimorphic for several traits [53, 54], *L. fuscus* is a species easily maintained in captivity that apparently has a weak dimorphic signature characterized by similar body sizes between sexes and by the absence of the male reproductive thumb spine observed in most leptodactylid frogs [53, 54]. A database comprising one possibly monomorphic and one dimorphic species grants the ideal experimental design for testing the role of testosterone in phenotypic integration because variation in dimorphic signals within *Leptodactylus* may reside in species-specific dynamics of the hormonal milieu. Identification of dimorphic patterns in *Leptodactylus podicipinus* will nurture comparisons between our two postulated hypotheses (Fig. 1b and c), while the advent of masculinized patterns in the monomorphic *L. fuscus* elicited by testosterone manipulation will validate the developmental role of this hormone in phenotypic integration of dimorphic traits in Anura. As a major outcome from this study, we use our results to propose a model for Tetrapoda that can explain the evolution of sexual dimorphism in 2D:4D and other digit ratios under the scenario of phenotypic integration.

## Methods

### Natural populations of *Leptodactylus podicipinus* and *L. fuscus*

**Fig. 2:**
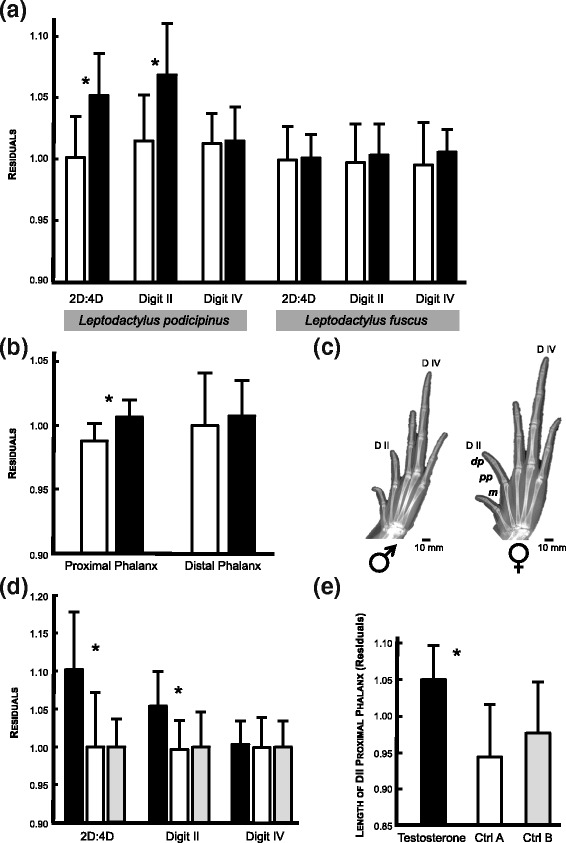
Comparisons between sexes and among treatments in *Leptodactylus frogs.* **a** Residuals against body size of digit ratios 2D:4D (2D:4D) and lengths of Digit II and Digit IV for males (*black*) and females (*white*) of *Leptodactylus podicipinus* and *L. fuscus*. **b** Residuals of phalangeal lengths (proximal and distal phalanges) of Digit II in males (*black*) and females (*white*) of *Leptodactylus podicipinus*.**c** X-ray images indicating bone segments measured in adult specimens (scale bar of 10 mm provided): DII = Digit II, D IV = Digit IV, dp = distal phalanx, pp. = proximal phalanx, m = metacarpal. **d** Results from experimental testosterone exposure of *Leptodactylus fuscus* tadpoles during development, indicating differences in 2D:4D and digit lengths between the testosterone-exposed group (*black*) and the two controls (*white*: Control A = vehicle solution only and *grey*: Control B = only water). **e** Osteological differences regarding the length of Digit II proximal phalanx between the group treated with testosterone (*black*) and the two controls (*white*: Control A = vehicle solution only and *grey*: Control B = only water). In all graphs, the columns correspond to mean values, and the vertical bars indicate standard errors; all measurements were taken in millimeters. Statistically significant differences (*p* < 0.05) are indicated by asterisks

The dataset of *L. podicipinus* comprised 373 adult specimens (191 females and 182 males) from herpetological collections that were sampled in localities from six different Brazilian states (Additional file 1: Figure S1). The specimens measured were part of collection lots comprising animals collected from 1970 to 2008 and deposited at the following Brazilian collections: DZSRP-UNESP; MZUSP-USP; Herpetological Collection Célio F.B. Haddad; collection from the Laboratory of Taxonomy, Systematic and Evolution of Neotropical Anurans at UFMG. The dataset of *L. fuscus* comprised 49 adult specimens (25 females and 24 males) sampled in two localities at different Brazilian states (Additional file 1: Figure S1). We sampled different sites to minimize population bias, since the presence and magnitude of sexual dimorphism in 2D:4D may vary among populations [10, 15, 39]. The lengths of Digits II and IV were measured in the hind limbs (right and left sides) using a digital caliper (Mitutoyo, Illinois, USA, accuracy of 0.01 mm), which was also used to measure the Snout-Vent Length (SVL) of each specimen. All measurements were taken by the same person (L.L.). After identifying which digit was sexually dimorphic in natural populations of *Leptodactylus* frogs (see results), we obtained digital X-rays (Faxitron LX-60) from adult specimens and measured the length of digit elements (Fig. 2c) in the dimorphic digit (see results) using the software ImageJ [55]. All morphological traits (lengths of Digit II, Digit IV, phalanges of Digit II, and SVL) were log10 transformed prior to calculations, and the digit ratio 2D:4D was calculated individually for front and hind limbs, by dividing length of Digit II by the length of Digit IV, as commonly reported in the current literature [11]. The effect of body size on digit lengths was corrected through a regression analysis [56] of each variable on SVL, and then the statistical analyzes were performed from extracted residuals.

### Treatments with testosterone levels in *Leptodactylus fuscus*

Tadpoles of *L. fuscus* comprised two clutches collected on the campus of University of São Paulo at Ribeirão Preto (state of São Paulo, Brazil) in December 2014. Animals were collected under ICMBio permit 32,862–1, following protocols from IUCN [57], and the experimental procedures were implemented according to the Animal Research Committee of University of São Paulo (CEUA/USP). Tadpoles from the two clutches of *L. fuscus* were mixed in the lab and randomly distributed into six aquariums (4 L water each), corresponding to duplicates for each of the three following conditions: 1) treatment with testosterone hormone (17α-methyltestosterone, IcquaImagem) and vehicle solution (absolute ethanol); 2) Control A, with only the vehicle solution (absolute ethanol); 3) Control B, comprising animals raised only in water, without exogenous testosterone or vehicle solution. Conditions 1 and 2 consisted of tadpoles first immersed in tanks with 1 l of water for 12 h, and then at the testosterone treatment 500 μg of 17α-methyltestosterone was diluted in absolute ethanol (adjusted to 0.05% ethanol /1 L), while in Control A, the vehicle solution alone (ethanol = 0.05% ethanol/1 L) was added to the water; in Control B (only water), the tadpoles were simply maintained at their initial condition (tanks with 1 l of water). Testosterone concentrations and treatment procedures followed the literature available for sex reversals [58–60]. Experiments started at developmental stage 28, which is characterized by early bud limb development according to the Gosner developmental table for anurans [61], and ended at stage 42, which comprises the beginning of the metamorphic stage [61]. Every 48 h, the procedures reported for the testosterone treatment and Control A were repeated (i.e., solutions at the aforementioned concentrations were added to the aquaria water). After reaching stage 42 [61], solutions (testosterone + vehicle or vehicle only) were not added to the water anymore, and the animals were maintained in the aquaria until the end of metamorphosis (post-metamorphic stage). The developmental windows chosen to apply the testosterone treatments embrace major events of autopodium and digit development in anurans, and therefore represent a time frame likely sensitive to hormonal manipulations, especially because gonadal development occurs at these same developmental stages [45, 61–63]. At the post-metamorphic stage, the animals were killed using an overdose of anesthetic lidocaine, and specimens were fixed in formalin (15%) and ethanol (70%).

Measurements in post metamorphic frogs were performed in digital photos using ImageJ software [55]. The same traits measured in adult *Leptodactylus* specimens from natural populations were also obtained in the *L. fuscus* juveniles (24 individuals treated with testosterone, 20 individuals in the Control A group, and 25 individuals in the Control B group): lengths of Digits II and IV of the hind limbs (right and left sides) and the Snout-Vent Length (SVL). Phalangeal lengths of Digit II were measured in post-metamorphic frogs as corresponding to the distance between phalangeal calluses [64]. The acquisition and processing of morphological data in post-metamorphic frogs were performed likewise to the data from adult specimens (see details of these procedures in the Methods section entitled ‘*Natural Populations of Leptodactylus podicipinus and L. fuscus’*).

### Statistical analyses

Statistical analyses were performed in R software [65]; graphics were designed in GraphPad Prism v.5 [66]. After confirming symmetry between the left and right sides using a paired *t*-test (see Additional file 1: Table S1) we performed all analyses using data from the right hind limb.

In adults from natural populations of *Leptodactylus* frogs, we first tested whether the two species studied were sexually dimorphic in the 2D:4D ratio and body size. We also tested which was the dimorphic digit to confirm whether dimorphism in these species was male-biased and to compare evidence for the hypotheses we postulated (Fig. 1b and c). A two-way ANOVA was performed separately for each species; the lengths of Digit II and Digit IV, the 2D:4D ratio, and the SVL were dependent variables, while sex and locality were included as factors. Subsequently, we applied a one-way ANOVA to the phalangeal lengths (dependent variable) using sex as a factor to test which digit elements explained the observed dimorphism.

To test the effect of testosterone levels on the establishment of 2D:4D ratios and body size during ontogeny, we obtained data for *Leptodactylus fuscus* at the post-metamorphic stage in animals raised at one of the following three conditions: 1) treated with testosterone in ethanol solution; 2) Control A and 3) Control B. The following morphological traits were compared among individuals raised under these three conditions using one-way ANOVAs: lengths of Digit II and Digit IV, lengths of the distal and proximal phalanges of Digit II, the 2D:4D ratio, and the SVL. Post hoc Bonferroni comparisons were applied.

In this study, we also tested for phenotypic integration between the digit ratio 2D:4D and body size in *Leptodactylus* frogs. We calculated a sexual dimorphism index (SDI) for the digit ratio (SDI-2D:4D) and for body size (SDI-SVL) for each sampled locality of the sexually dimorphic species (*L. podicipinus*, see results) following the current literature [67]. Spearman-correlation tests were then performed to test for significant associations between SDI-2D:4D and SDI-SVL in each locality. Statistically significant associations were interpreted as evidence for phenotypic integration between the two traits, likely mediated by multi-trait hormonal modulation during ontogeny.

## Results

### The male-biased 2D:4D dimorphic pattern is determined by the identity of the dimorphic digit

In natural populations of *L. podicipinus*, the digit ratio 2D:4D is sexually dimorphic (*F*_1, 366_ = 38.715, *p* < 0.001; Fig. 2a), while in *L. fuscus* it seems monomorphic (*F*_1, 46_ = 0.063, *p* = 0.801; Fig. 2a). The dimorphic pattern detected in *L. podicipinus* 2D:4D digit ratio is male-biased as follows: males exhibit larger relative ratios than females (Fig. 2a). Such differences between sexes are settled in the relative length of Digit II, which is significantly longer in males than in females of *L. podicipinus* (*F*_1, 366_ = 15.873, *p* < 0.001; Fig. 2a). The relative length of Digit IV, in contrast, does not differ between males and females of this species (*F*_1, 366_ = 1.407, *p* = 0.236; Fig. 2a). Between-sex differences in Digit II, instead of Digit IV, corroborate the ‘*hypothesis of identity of the dimorphic digit’*. We then used X-ray images to test which digit elements explain the sexually dimorphic pattern identified in *L. podicipinus* and found that males have a longer proximal phalanx of Digit II than females (*F*_1, 19_ = 6.633, *p* = 0.024; Fig. 2b and c).

We also evaluated the developmental role of testosterone in the establishment of 2D:4D digit ratios and body size. Specifically, we exposed tadpoles of *Leptodactylus fuscus*, a species monomorphic for 2D:4D digit ratios that is easily maintained in captivity, to increased concentrations of testosterone based on protocols of sexual reversion [58–60]. We used the following two controls: control A = addition of ethanol solution to the water, and control B = tadpoles raised in water without any vehicle/ethanol. The patterns resulting from experimental treatments with testosterone are coherent with the ‘*hypothesis of identity of the dimorphic digit*’, which has already been supported by data from adult specimens of *L. podicipinus* from natural populations. Recently metamorphosed individuals from the group treated with testosterone at the larval stage exhibited masculinized 2D:4D digit ratios (*F*_2, 66_ = 48.099, *p* < 0.001; Fig. 2d); the two control treatments produced similar phenotypes between each other (*p* = 0.140; Fig. 2d). Only Digit II responded to manipulations of hormonal levels and became longer in animals treated with testosterone (*F*_2,66_ = 42.979, *p* < 0.001; control groups did not differ from each other in post hoc comparisons *p* = 0.524; Fig. 2d). Testosterone had no effect on Digit IV (*F*_2,66_ = 0.3886, *p* = 0.5351; Fig. 2d). Similar to our observations in adults of *L. podicipinus*, the digital element that responded to variation in hormonal levels was the proximal phalanx of Digit II (*F*_2,27_ = 11.538, *p* < 0.001; control groups did not differ from each other in post hoc comparisons: *p* = 0.990; results from testosterone treatments differed from Control A (*p =* 0.018) and Control B (*p =* 0.025); Fig. 2e). Altogether, these results corroborate the hypothesis that a 2D:4D male-biased pattern of sexually dimorphic digit ratios derives from a taxon-specific change in the identity of the digit that is sensitive to the levels to sex steroids during ontogeny.

### Sexual dimorphism in body size is coupled with male-biased 2D:4D digit ratios in *L. podicipinus*, and testosterone induces masculinized body sizes and digit ratios in tadpoles of the monomorphic *L. fuscus*

**Fig. 3:**
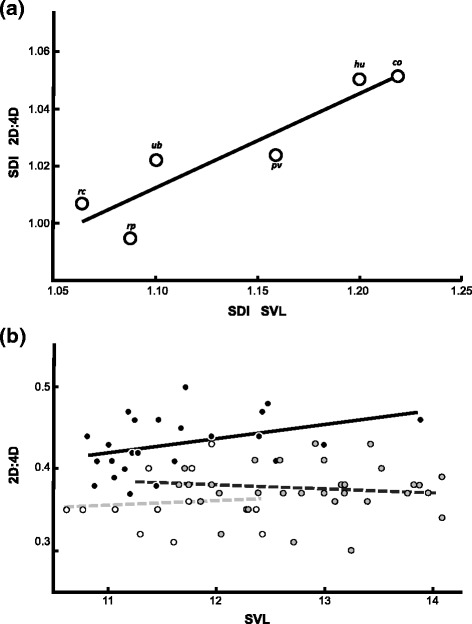
Phenotypic integration of body size and digit ratios in *Leptodactylus.* **a** Correlation between sexual dimorphism indexes (SDI) for the digit ratio 2D:4D and body size (SVL) for the six *L. podicipinus* populations. The localities are Humaitá, AM (*H*), Corumbá, MT (*C*), São José do Rio Preto, SP (*RP*), Uberlândia, MG (*Ub*), Rio Claro, SP (*RC*) and Porto Velho, RO (*PV*). **b** Correlation between body size (SVL) and 2D:4D digit ratios of *L. fuscus* tadpoles in three conditions: treated with testosterone (*black*), Control A (*white*) and Control B (*grey*); all measurements were taken in millimeters

Our results also provided evidence for phenotypic integration between 2D:4D ratios and body size, likely hormonally modulated by testosterone levels during larval development. The species *L. podicipinus* is sexually dimorphic for body size, and females are significantly bigger than males (*F*_1,366_ = 231.470, *p* < 0.001). Congruent with our findings for *L. fuscus*, digit ratios*,* males and females of this species do not differ in body size (*F*_1,46_ = 1.832, *p* = 0.182). In addition, the sexual dimorphism identified in *L. podicipinus* differed in magnitude among localities (see Additional file 1: Table S2), and individuals from localities characterized by larger values of the Sexual Dimorphism Index in Digit Ratios (SDI-2D:4D) also exhibit larger values of the Sexual Dimorphism Index in Body Size (SDI-SVL) (*Spearman correlation*: *r* = 0.9429, *p* = 0.017; Fig. 3a), an association that suggests phenotypic integration among traits. The role of testosterone acting as a modulator of phenotypic integration was corroborated in the experimental treatments performed with *L. fuscus* tadpoles. Individuals treated with higher testosterone concentrations during development were significantly smaller than both controls (*F*_2,66_ = 14.361, *p* < 0.001). Significant correlations between SVL and the digit ratio 2D:4D were only identified among individuals treated with testosterone (*r* = 0.397, *p* = 0.027; Fig. 3b), an association not detected in the controls (Control A: *r* = 0.077, *p* = 0.405; Control B: *r* = −0.120, *p* = 0.252). Altogether, our databases suggest that testosterone is a major regulator of the establishment of female-biased body size and male-biased 2D:4D digit ratios in *Leptodactylus* frogs and that it is a very likely promoter of phenotypic integration in Anura.

## Discussion

**Fig. 4:**
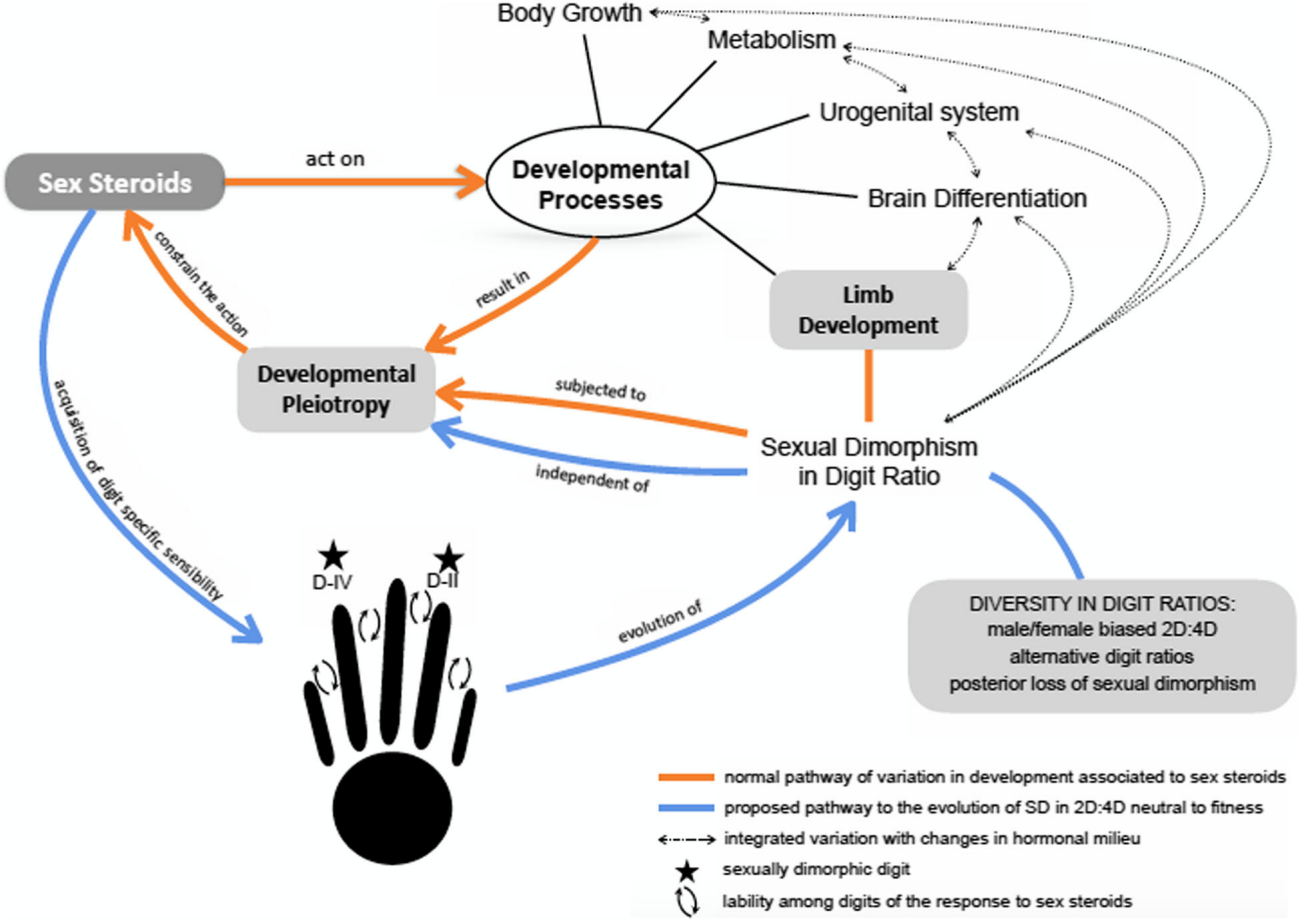
Hypothetic model of the evolution of digit ratio dimorphism modulated by multi-trait hormonal effects on developmental pathways. Acquisition of higher sensitivity to sex steroids in specific digits (indicated by stars, Digit II or IV) is initially independent of hormonal concentrations per se (see *blue arrows*), and the identity of dimorphic digits seems labile among tetrapod lineages (*small black arrows* among digits). The advent of sexual dimorphism in digit ratios evolves independently of variation in the hormonal milieu (*blue arrows*), and such trajectory results on a diversity of patterns of digit ratio dimorphism in Tetrapoda. Once the sensibility of a dimorphic digit to steroids is present in the lineage, the length of the digit (and consequently the digit ratio) changes in response to variations in hormonal concentrations and together with others traits that are also hormonally-regulated (*doted black arrows*).Multi-trait effects of circulating steroids during development (*orange lines and arrows*) establish correlated variation between digit ratios and other dimorphic traits

Dimorphic patterns in the digit ratio 2D:4D, coupled with sexual dimorphism in other phenotypic traits, such as body size, provide broad evidence for ontogenetic phenotypic integration mediated by steroid hormones circulating during development [8, 13–43]. Hormonal pleiotropic effects are often responsible for constrains at different biological levels [3–7], which may persist along the entire ontogeny [68, 69]. Evolutionary processes that involve a dissociation between variation in the hormonal milieu and the response capacity of specific tissues apparently consent changes in hormonally regulated traits that disregard variation in the hormone concentrations per se, circumventing possible pleiotropic effects derived from changes in the hormonal milieu [5, 6]. Here, we propose that the initial establishment of a sex-differential sensibility of digit tissues to circulating hormones may not depend on primary milieu changes per se. The observed integration between digit ratios and other sexually dimorphic traits [8, 13–43] would therefore be secondary, assuming a coalesced modulation by hormonal concentrations after the initial advent of a differential digit sensitivity to circulating hormones. Based on our results from *Leptodactylus* frogs, together with current literature, we propose a model to explain the origin and evolution of female and male-biased patterns of sexual dimorphism in 2D:4D digit ratios that is illustrated in Fig. 4. Our model states that, once the dimorphism in digit ratios arises during evolution (Fig. 4, blue arrows), it becomes phenotypically integrated with other sexually dimorphic traits (Fig. 4, orange arrows) because changes in the hormonal milieu that regulates different developmental processes will affect traits that ancestrally were not correlated. However, the occurrence of sexual dimorphism in digit ratios and the direction of its patterns (i.e., male-biased or female-biased) depend primarily on the existence of a given digit being more sensitive to hormone concentrations than the others (Fig. 4, blue arrows). Such difference in hormone sensitivity would be established by increased concentrations of steroid receptors in that digit and, since it is possible that this initial change in digit sensitivity to steroids did not directly affect survival or reproductive success, the advent of enhanced sensitivity of digit development to steroids might have been fitness-independent on its origin or, in other words, neutral to fitness [6].

Illustrating the pleiotropic effects of testosterone during development, our results shed light on the mechanistic basis of body size sexual dimorphism in anurans: testosterone treatments in the monomorphic *L. fuscus* rescued patterns of female-biased body sizes and male-biased digit ratios identified in natural populations of *L. podicipinus*, and body size was correlated to digit ratios in hormone-treated tadpoles. Our results agree with current literature [3, 44] and indicate that testosterone may play a major role in the determination of female-biased body size dimorphism in Anura, an association also described in other vertebrate lineages, as Squamata [44]. Further investigation on hormonally mediated tradeoffs between early sexual maturity and survival in natural populations seems promising, as it is possible that testosterone represses growth in anuran males as a collateral effect of early maturation [44, 46–50].

The contrast between an apparent absence of sexual dimorphism in 2D:4D digit ratios and body size in adults of *L. fuscus* from natural populations and the testosterone-induced phenotypic changes in both traits on *L. fuscus* tadpoles deserves a more detailed discussion. Although sexual dimorphism has not been detected in digit ratios or body size of *L. fuscus* adults, our experimental approach provides evidence for the capacity of tissue responses to sex steroids in the species. One possible explanation is that the circulating testosterone concentrations in *L. fuscus* tadpoles are not high enough to surpass the threshold necessary for triggering dimorphic patterns, despite the tissue capacity for responding to hormonal variation. When testosterone is present at higher concentrations, as those applied in our experimental treatments (see also [58–60]), this threshold is surpassed, and integrated variation in phenotypic traits modulated by the hormonal milieu is then observed. Future studies characterizing circulating steroid levels in tadpoles and clutch foams of *Leptodactylus* may clarify if different patterns of sexual dimorphism between the two *Leptodactylus* species studied here reflect variation in hormonal developmental environments.

The correlated variation between body size and digit ratios in adults of *L. podicipinus* and testosterone-treated *L. fuscus* tadpoles has been interpreted here under a model where phenotypic integration could be established through indirect interactions during development mediated by the sharing of a common hormonal environment along specific developmental windows. In *Leptodactylus,* male-biased 2D:4D digit ratios apparently originated from a differential sensibility of Digit II developing tissues to circulating testosterone (*Hypothesis of changes in the identity of the dimorphic digit*, see Fig. 1c), instead of Digit IV being sensitive to hormones, as described in female-biased species and predicted by our Hypothesis I (*Hypothesis of sex-specific digit responses*, see Fig. 1b). The advent of increased sensitivity in one specific digit could evolve through changes in steroid receptors concentrations in specific phalanges of that digit (see [8, 9]). Once established, the identity of the digit that is sensitive to steroids apparently becomes inalterable in that given organism, and experimental manipulations of sex steroids modify the magnitude of digit ratio dimorphism and may originate sex-bias, but never change the taxa-specific pattern or the identity of the dimorphic digit [10, 32, 36, 37, 40, 70, 71]. Along with such observation, several species of primates, birds, frogs and lizards experienced secondary losses of sexual dimorphism in digit ratios that were not coupled with loss of sexual dimorphism in other traits [10, 34, 38, 43]. Such information assemblage reinforces our proposition that the identity of the digit that becomes dimorphic due to differential sensitivity to circulating steroids is not dependent of the hormonal developmental environment per se, even if the associations between sexual dimorphism in digit ratios and other traits reflect sex-steroid pleiotropy [8, 9]. If true, sexual dimorphism in digit ratios might not be the direct target of selection, unless dimorphic digits reflect differential performance between males and females (see [42]). Consequently, the origin of sexual dimorphism in a given digit length might be neutral to fitness [6]; because sexually dimorphic patterns in digit ratios would initially be hidden from selection, the system would be permissive to considerable variation among lineages in the patterns and magnitudes of the dimorphism (Fig. 4). This ‘fitness-independent model’ is not only applicable to explain the evolution of male and female-biased 2D:4D digit ratios [10, 41, 43], but may also elucidate the occurrence of sexual dimorphism in other digit ratios, such as 3D:4D and 2D:3D [40, 41, 43], contributing also to the elucidation of other processes, for example involving the loss of sexual dimorphism in digit ratios in species that remain sexually dimorphic for other traits [10, 34, 38, 43]. Indeed, our model predicts that further research using other tetrapod lineages will find additional evidence supporting the alternative digit ratio patterns just mentioned.

## Conclusions

The difference between lineages that exhibit a male-biased 2D:4D and those characterized by a female-biased 2D:4D resides on the identity of the dimorphic digit responding to steroids during development. In lineages such as Anura, which exhibit male-biased 2D:4D, it is the Digit II, instead of IV, that is sexually dimorphic and steroid-responsive. Dimorphism in digit ratios is developmentally correlated with variation in body size in anurans, an association identified in *Leptodactylus* natural populations and validated by experiments exposing developing tadpoles to increased testosterone concentrations. We provide evidence that testosterone plays a major role in the establishment of such phenotypic integration, and likely rules the evolution of female-biased body size dimorphism in Anura. We combined our findings with current literature to propose an explanatory model for the origin and evolution of the sexual dimorphism in digit ratios. Specifically, we propose that a first event leading to the advent in a given digit of increased tissue sensitivity to steroids would then engender phenotypic integration of digit ratios with other sexually dimorphic traits. Such model explains the apparent lack of specific functions associated to variation in digit ratios, the diversity of dimorphic patterns evolving in different tetrapod lineages, and the profusion of alternative digit ratio dimorphism in tetrapods (3D:4D, 2D:3D). The posterior establishment of phenotypic integration between digit ratios and other sexually dimorphic traits would occur through multi-trait hormonal effects during development. In other words, the advent of digit sensitivity that results in dimorphism of digit ratios would constitute a discrete event, which is not necessarily connected to its developmental association with other dimorphic traits. Extension of current research programs to incorporate the proposed model might produce the following outcomes: i) identification of more lineages exhibiting male or female-biased 2D:4D dimorphism; ii) inclusion of alternative digit ratios (3D:4D, 2D:3D) in studies investigating evolution of intersected dimorphism among traits; iii) comprehension of cases where dimorphism in digit ratios have been lost while sexual dimorphism remained present.

## Additional file

Additional file 1: Table S1. Results of the symmetry tests between right and left measurements in *Leptodactylus* frogs; *d.f.* = degrees of freedom. **Table S2.** Mean and standard deviation for Sexual Dimorphism Indexes (SDI) of SVL and the 2D:4D ratio, calculated for different localities of *Leptodactylus podicipinus*; codes within parentheses indicate the Brazilian state of each locality. **Figure S1.** Localization of each natural population sampled for measurements in adults. Localities where both species were collected: 1) Humaitá (AM) and 6) São José do Rio Preto (SP). Localities where *L. podicipinus* was collected: 2) Porto Velho (RO), 3) São José do Rio Claro (MT), 4) Corumbá (MS), and 5) Uberlândia (MG). (PDF 355 kb)

## Abbreviations

2D:4D: Ratio between digits II and IV lengths
SDI: Sexual dimorphism index
SDI-2D:4D: Sexual dimorphic index for the digit ratio 2d:4d
SDI-SVL: Sexual dimorphism index for the snout-vent length
SVL: Snout-vent length
