# Supplementary material for: Phenotypic integration mediated by hormones: associations among digit ratios, body size and testosterone during tadpole development
Source: BMC Evol Biol. 2017 Aug 2;17:175. doi: 10.1186/s12862-017-1021-0 (PMC5541650; doi:10.1186/s12862-017-1021-0)
Supplement: Additional file 1: Table S1. — Results of the symmetry tests between right and left measurements in Leptodactylus frogs; d.f. = degrees of freedom. Table S2. Mean and standard deviation for Sexual Dimorphism Indexes (SDI) of SVL and the 2D:4D ratio, calculated for different localities of Leptodactylus podicipinus; codes within parentheses indicate the Brazilian state of each locality. Figure S1. Localization of each natural population sampled for measurements in adults. Localities where both species were collected: 1) Humaitá (AM) and 6) São José do Rio Preto (SP). Localities where L. podicipinus was collected: 2) Porto Velho (RO), 3) São José do Rio Claro (MT), 4) Corumbá (MS), and 5) Uberlândia (MG). (PDF 355 kb) [file 12862_2017_1021_MOESM1_ESM.pdf]

Supplementary Information

Phenotypic integration mediated by hormones: associations among digit ratios, body size and testosterone during tadpole development.

Leandro Lofeu, Renata Brandt & Tiana Kohlsdorf

Table S1: Results of the symmetry tests between right and left measurements in *Leptodactylus* frogs; *d.f.* = degrees of freedom.

	Species	Trait	*d.f.*	*t-value*	*p-value*
Natural Populations	*L. podicipinus*	Digit II	367	0.122	0.902
		Digit IV	361	0.228	0.819
		2D:4D	361	-2.230	0.817
	L. fuscus	Digit II	48	0.920	0.322
		Digit IV	48	0.361	0.719
		2D:4D	48	0.504	0.616
Testosterone treatment	*L. fuscus*	Digit II	68	-0.905	0.370
		Digit IV	68	0.950	0.924
		2D:4D	68	-0.975	0.334

Table S2: Mean and standard deviation for Sexual Dimorphism Indexes (SDI) of SVL and the 2D:4D ratio, calculated for different localities of *Leptodactylus podicipinus*; codes within parentheses indicate the Brazilian state of each locality.

Locality	SDI – 2D:4D ratio	SDI - SVL
Humaitá (AM)	1.0503±0.0419	1.1999±0.0566
Porto Velho (RO)	1.0238±0.0271	1.1588±0.0436
São José do Rio Claro (MT)	1.0069±0.0309	1.0638±0.0493
Corumbá (MS)	1.0514±0.0457	1.2187±0.0538
São José do Rio Preto (SP)	0.9947±0.0349	1.0874±0.0405
Uberlândia (MG)	1.0222±0.0307	1.1001±0.0361

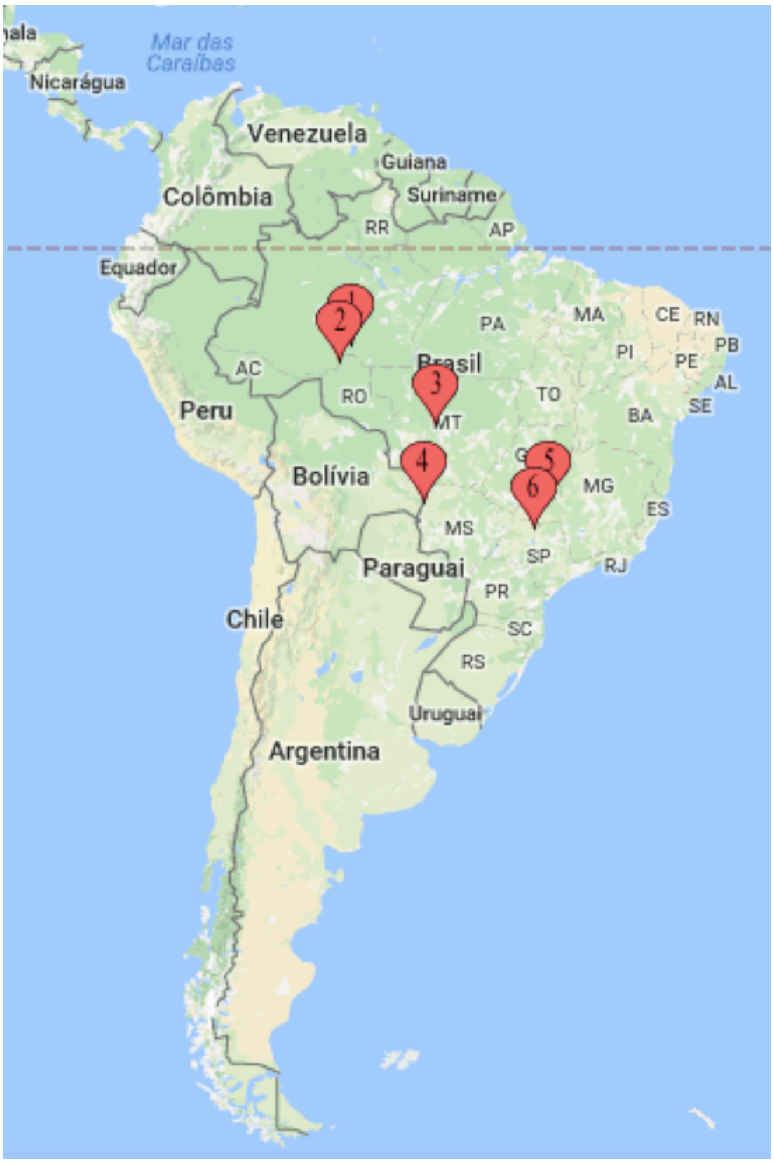

Figure S1: Localization of each natural population sampled for measurements in adults. Localities where both species were collected: 1) Humaitá (AM) and 6) São José do Rio Preto (SP). Localities where *L. podicipinus* was collected: 2) Porto Velho (RO), 3) São José do Rio Claro (MT), 4) Corumbá (MS), and 5) Uberlândia (MG).
